# Effects of sympathectomy on myocardium remodeling and function

**DOI:** 10.6061/clinics/2021/e1958

**Published:** 2021-01-11

**Authors:** Maurício Rodrigues Jordão, Fernanda G. Pessoa, Keila C.B. Fonseca, Fernando Zanoni, Vera M.C. Salemi, Leandro E. Souza, Orlando N. Ribeiro, Fábio Fernandes, Maria Claudia Irigoyen, Luiz Felipe P. Moreira, Charles Mady, Felix Jose Alvarez Ramires

**Affiliations:** Instituto do Coracao (InCor), Hospital das Clinicas (HCFMUSP), Faculdade de Medicina, Universidade de Sao Paulo, Sao Paulo, SP, BR

**Keywords:** Autonomic Modulation, Sympathectomy, Heart Failure, Physiology

## Abstract

**OBJECTIVES:**

To evaluate the effects of sympathectomy on the myocardium in an experimental model.

**METHODS:**

The study evaluated three groups of male Wistar rats: control (CT; n=15), left unilateral sympathectomy (UNI; n=15), and bilateral sympathectomy (BIL; n=31). Sympathectomy was performed by injection of absolute alcohol into the space of the spinous process of the C7 vertebra. After 6 weeks, we assessed the chronotropic properties at rest and stress, cardiovascular autonomic modulation, myocardial and peripheral catecholamines, and beta-adrenergic receptors in the myocardium. The treadmill test consisted of an escalated protocol with a velocity increment until the maximal velocity tolerated by the animal was reached.

**RESULTS:**

The bilateral group had higher levels of peripheral catecholamines, and consequently, a higher heart rate (HR) and blood pressure levels. This suggests that the activation of a compensatory pathway in this group may have deleterious effects. The BIL group had basal tachycardia immediately before the exercise test and increased tachycardia at peak exercise (*p*<0.01); the blood pressure had the same pattern (*p*=0.0365). The variables related to autonomic modulation were not significantly different between groups, with the exception of the high frequency (HF) variable, which showed significant differences in CT *vs* UNI. There was no significant difference in beta receptor expression between groups. There was a higher concentration of peripheral norepinephrine in the BIL group (*p*=0.0001), and no significant difference in myocardial norepinephrine (*p*=0.09).

**CONCLUSION:**

These findings suggest that an extra cardiac compensatory pathway increases the sympathetic tonus and maintains a higher HR and higher levels of peripheral catecholamines in the procedure groups. The increase in HF activity can be interpreted as an attempt to increase the parasympathetic tonus to balance the greater sympathetic activity.

## INTRODUCTION

Heart failure (HF) is estimated to affect 1% to 2% of the population, reaching 10% in those over 70 years of age, and it’s prevalence is growing rapidly in developed countries (1). Moreover, despite the fact that spending for HF treatment is high, mortality remains high. The publication of the benefits of beta-blockers in heart failure has led to a relative risk reduction in mortality of approximately 30% (2). Furthermore, other possibilities for interventional or device-based approaches through the modulation of autonomic activity, such as spinal cord stimulation, vagus nerve stimulation, baroreflex activation therapy, renal sympathetic nerve denervation, and cardiac sympathetic denervation (CSD), have also been proposed (2). Currently, vagus nerve and spinal cord stimulation has shown no benefits, as reported in the INOVATE-H (3) and Defeat-HF (4) trials, the two major studies on each topic, respectively. Regarding baroreflex activation therapy, Zile et al. (5) showed some benefit and safety. Renal sympathetic nerve denervation therapy is still under investigation, (6) but has already been found to be safe. Furthermore, renal sympathetic nerve denervation therapy has been shown to improve left ventricular systolic function, and reduce the mean heart rate (HR) without altering renal function. CSD has also been tested in patients with heart failure and has been proven to be both feasible and safe. Although there was no difference in VO_2_ peak in brain natriuretic peptide levels or muscle sympathetic activity, patients in the intervention group showed significant improvement in the 6-minute walk test and quality of life (7). This study raised interesting questions and possibilities. However, the effects of CSD on cardiac structure and function have not been adequately studied in a physiological scenario (8,9).

The present study was conducted to evaluate the physiological effects of sympathectomy on the myocardium in an experimental rat model. Specifically, the study assessed the impact of sympathectomy on myocardial beta receptors, myocardial and peripheral catecholamines, structural and functional changes in the heart, response to physical exercise, and also evaluated autonomic function.

## MATERIAL AND METHODS

Male Wistar rats 8 weeks of age were divided into three groups: control (CT; n=15), left unilateral sympathectomy (UNI; n=15), and bilateral sympathectomy (BIL; n=31). The study was designed such that the animals underwent electrocardiography, echocardiography, treadmill testing, evaluation of autonomic function, myocardial beta receptors, myocardial and peripheral catecholamines, and myocyte size evaluation 6 weeks after the surgical procedure.

### Animal model

For the surgical procedure, the rats were anesthetized in a prone position with isoflurane (3.0 vol% for induction and 1.5 vol% for maintenance) and oxygen (100%). After palpation of the spinous process of the C7 vertebra, a needle was inserted, attached to a 1.0-mL syringe, to a sagittal paramedian plane, advancing in the posteroanterior direction of the spinous process. When the needle tip lost contact with the vertebral body (a signal that it had passed the anterior aspect of the vertebral body), 0.5 mm was retracted, and 0.2 mL of absolute alcohol was injected (10). Clinical confirmation was made by the observation of ipsilateral nonreversible ptosis. In the BIL group, due to high mortality (77%), animals that died during the procedure or up to 4 hours afterwards were replaced. The protocol was performed in accordance with the recommendations of The Brazilian College of Experimental Animal Studies (Colégio Brasileiro de Experimentação Animal 2013) (11) and the Guide for the Care and Use of Laboratory Animals (National Research Council 2011) (12). The study protocol was reviewed and approved by the Ethics Committee of the Heart Institute and the University of São Paulo Medical School.

### Electrocardiography

After 6 weeks, electrocardiography for HR, PR, and QTc interval analysis was performed with the rats under general anesthesia with a combination of intraperitoneal ketamine (50 mg/kg) and xylazine (10 mg/kg). The QT interval (ms) and HR (RR) were measured, and the QTc interval was evaluated using the Bazett formula (QTc=QT/√RR). Electrocardiography was performed using the Dixtal-Eletropage^®^ device connecting the electrodes on the front and back paws of the animal and respecting the references: right arm, left arm, left leg, and right leg. The derivations DI, DII, DIII, aVR, aVL, and aVF were performed with a gain of 2N and a velocity of 50 mm/s. The paper used was the ECG Recording Paper Series 4700AH from Hewlett-Packard.

### Echocardiography

Immediately after electrocardiography, with the rats still anesthetized, transthoracic mode M, 2-dimensional echocardiography was performed using the Acuson, Sequoia model 512, with a 9-mm transducer and a frequency of 13 mHz. The patterns of regional and global contraction were evaluated in real time in the parasternal long and short axes of the left ventricle (LV). The cardiac systolic and diastolic dimensions were assessed using the M mode. Left ventricular function was evaluated by the ejection fraction (Cube method) and fraction-shortening analysis. The study was performed per standardization in rats, and normal echocardiograms were obtained in the control group.

### Treadmill test

Two days after electrocardiography and echocardiography, the rats underwent cannulation of the right carotid artery while under inhaled anesthesia using a mask in a volume of 3 L of oxygen with 2% isoflurane. This procedure allowed measurements of autonomic modulation, blood pressure (BP), and HR during the treadmill test. The treadmill test consisted of an escalated protocol with a velocity increment of 0.3 km/h every 3 minutes until the maximal velocity tolerated by the animal was reached. Exhaustion was determined at the point when the rats were no longer able to overcome the treadmill velocity, at which point, the peak exercise BP and HR were measured (13). The length and exercise time were also measured, as well as the BP and HR at rest, before starting the treadmill test.

### Evaluation of autonomic modulation

The HR and systolic blood pressure (SBP) variability were measured in the time domain (variance) and the frequency domain using the fast Fourier transform. With this method, the pulse interval time series and SBP were divided into segments of 512 beats with a 50% overlap. A spectrum was obtained for each of the segments, and the oscillatory components of the spectra were measured in two frequency bands: low frequency (LF: 0.20 to 0.75 Hz), representing predominantly sympathetic modulation, and high frequency (HF: 0.75 to 3 Hz), representing predominantly parasympathetic modulation. The spectrum power was calculated for each component of low frequency and high frequency bands integrating the spectra using Cardioseries 2.4 software. The segments that showed very slow oscillations (<0.2 Hz) were considered nonstationary and were dropped from the analysis. The spontaneous baroreflex sensitivity was obtained from index α, by analyzing the temporal and linear correlation between the absolute value of LF for the pulse interval (PI) and SBP (14,15). The PI variables were evaluated, including the standard deviation (PI SD) and variance (PI VAR). The SBP variance (SBP VAR), and absolute values of LF and HF were also evaluated. The analysis was performed just before the treadmill test.

### Myocardial and peripheral catecholamines

A commercial ELISA kit (Labor Diagnostika Nord) was used to evaluate the levels of peripheral adrenaline and noradrenaline. The reaction was performed in 96-well plates according to the manufacturer's instructions. The myocardial catecholamines were analyzed by high performance/pressure liquid chromatography.

### Quantitative real time PCR analysis for β receptors 

The apex and base from frozen hearts were used for gene expression analysis. Following total RNA isolation, including DNase treatment with Turbo DNA-Free^®^ (Ambion-The RNA Company), the RNA was reverse transcribed using SuperScript^®^ II Reverse Transcriptase (Invitrogen^®^). Quantitative real time-PCR was performed with the StepOnePlus^®^ Real-Time PCR System (Applied Biosystems, EUA) using the SYBR green method. The primers used are depicted in Table 1 (16-18).

### Myocyte size

The myocyte size was measured by morphometry in hematoxylin and eosin stained tissue; longitudinal sections were analyzed where the nuclei were oval and centralized. The diameter was then measured in μm with the aid of an optical microscope coupled to a computer with the program QWIN Image Processing and Analysis Software (Leica Microsystems Cambridge Ltd).

### Statistical analysis

The Shapiro-Wilk test was used for normality testing. For normally distributed variables, two-tailed one-way analysis of variance (ANOVA) was performed for the three group comparisons, and the post-hoc Tukey test was used for multiple comparisons. The data are presented as mean±standard deviation. For non-normally distributed variables, we applied the Kruskal-Wallis and Dunn tests for the differences, with the results presented as median and 25th to 75th percentiles. The level of significance was 5%.

## RESULTS

A total of 15 animals were included in the CT and UNI groups, and 31 animals were included in the BIL group. The UNI group had a 13% mortality rate, leaving 13 animals, and the BIL group had a 77% mortality (31 animals operated on) leaving seven animals. There was no mortality in the CT group. For each analyzed variable, all the survivors were included, except for the maximum aerobic treadmill test and autonomic modulation evaluation, where only five animals each from the CT and UNI groups, and seven animals from the BIL group were included.

### Electrocardiogram

At resting ECG, there were no significant differences in HR (beats per min [bpm], CT: 287±34; UNI: 308±35; BIL: 300±33; *p*=0.30), QTc (ms, CT: 207±20; UNI: 213±29; BIL: 218±16; *p*=0.85), and PR interval (ms, CT: 115±10; UNI: 110±11; BIL: 125±10; *p*=0.18) between the groups.

### Echocardiogram

The left ventricular end-diastolic diameter (LVDD) was slightly larger in the CT group, but with no significance (*p*=0.09). A similar response was observed for left ventricular end-systolic diameter (LVSD), with a slightly larger LV in the CT group, with no significance (*p*=0.06). The LV thickness (septum and free wall) and the left atria (LA) were not significantly different between the three groups. Moreover, we found no significant differences in LV systolic function between the three groups (*p*=0.18). The echocardiogram results are summarized in Table 2.

### Treadmill test

In the treadmill test, the variables for time, maximum velocity, and distance were not significantly different among the three groups (Table 3).

**Figure 1:**
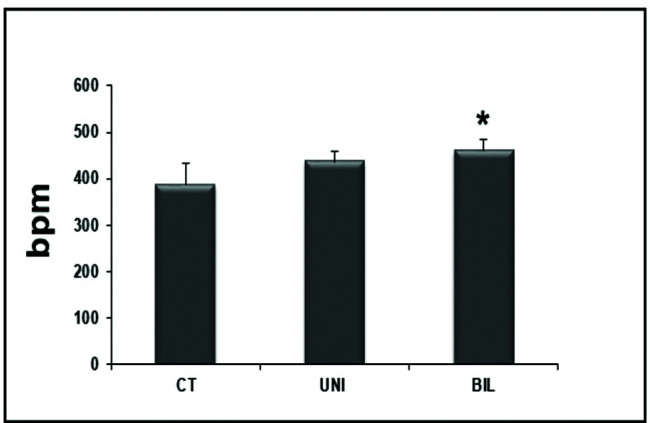
Resting HR with significant P-values in BIL *vs* CT (*p=0.03).

**Figure 2:**
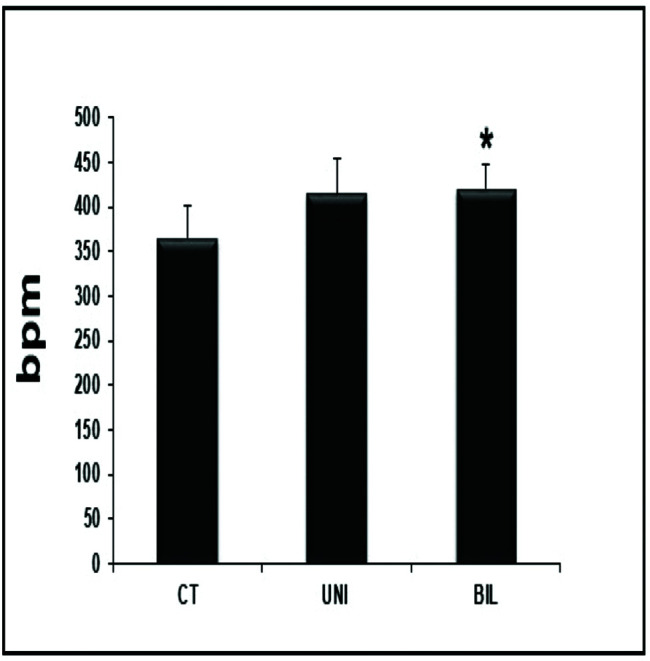
HR at peak exercise with significant P-values comparing BIL *vs* CT (*p=0.004).

Interestingly, the BIL group had higher values in the HR evaluation immediately before the exercise test compared to the controls (*p<*0.05) (Figure 1). At peak exercise, the BIL group sustained the HR with higher values compared to the CT group (*p*<0.01) (Figure 2).

In the analysis of SBP at rest compared to the SBP of the controls (*p*=0.02) and with UNI (*p*=0.04), the BIL group had higher levels (Table 4). The diastolic blood pressure (DBP) was also higher in the BIL group, although with no statistical difference (*p*=0.11) (Table 4). The mean arterial pressure (MAP) also was higher in the BIL group, although with no statistical difference (*p*=0.07). Similarly, immediately after exercise, the BIL group had higher SBP, DBP, and MAP than the controls (*p*<0.05, *p*=0.04, and *p=*0.04, respectively) (Table 5).

### Evaluation of autonomic modulation

**Figure 3:**
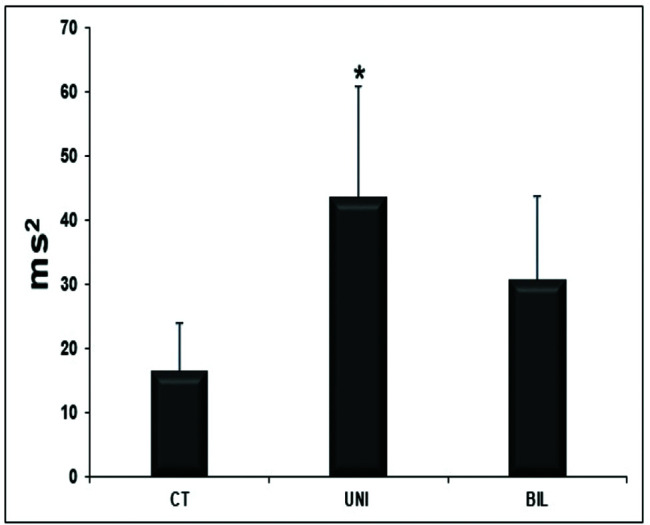
High frequency variable of autonomic modulation (*p<0.05, CT *vs* UNI)

The variables related to autonomic modulation showed no statistical significance (Table 6), except for the HF variable, which had significantly higher values in the UNI group than in the controls (Figure 3).

### Myocardial and peripheral catecholamines

**Figure 4:**
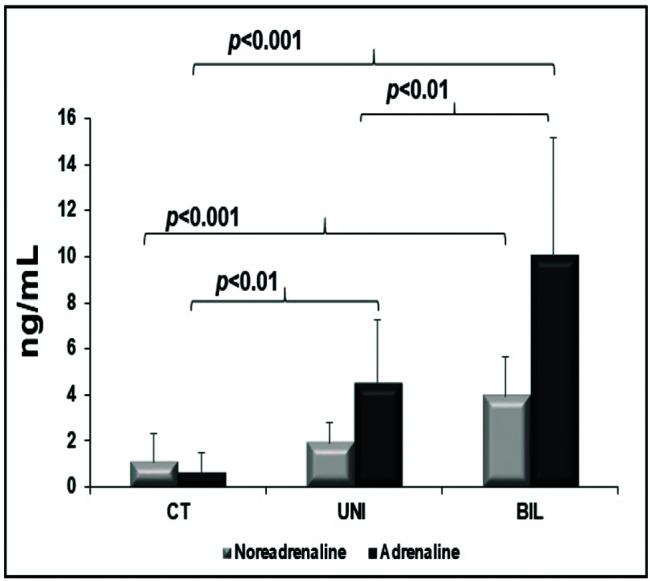
Peripheral catecholamines.

Myocardial catecholamines showed no statistical differences (myocardial adrenaline [pg/mL], CT: 12 [8-41]; UNI: 12 [12-12]; BIL: 12 [12-12]; *p*=0.2, and myocardial noradrenaline [pg/mL], CT: 1046±863; UNI: 375±464; BIL: 1078±1485; *p*=0.09). The serum adrenaline concentration was higher in the BIL group than in all the other groups and was higher in the UNI than in the control group. The serum concentration of noradrenaline was also higher in the BIL group compared to that in the controls (Figure 4)**.**

### β receptors

**Figure 5:**
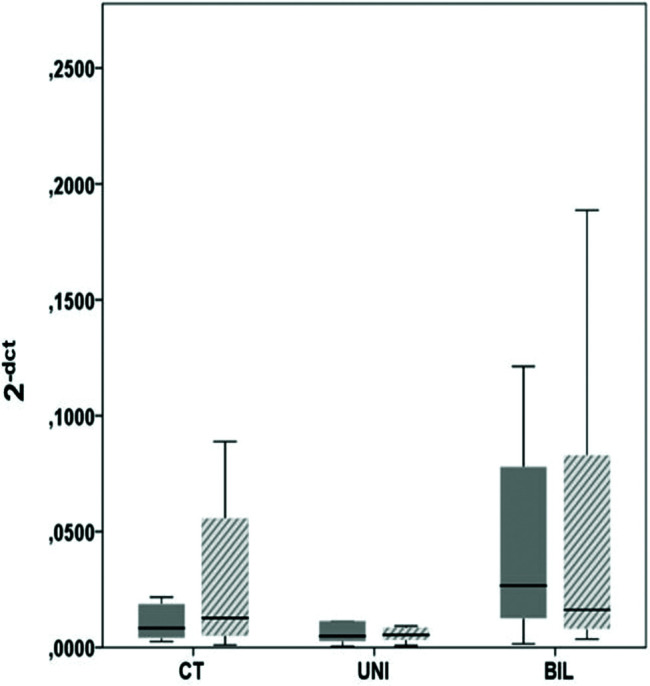
β1 receptors at the apex and base.

**Figure 6:**
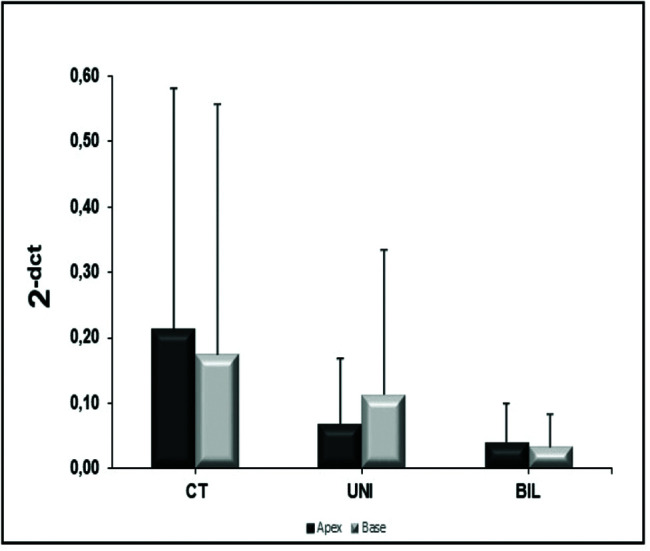
β2 receptors at the apex and base.

In the analysis of the β1 receptors in the LV, there was no significant difference between groups at the apex (*p*=0.09) or the base (*p*=0.11) (Figure 5). However, the BIL group had absolute double expression at the apex, despite the lack of a statistical difference. In the analysis of β2 receptors at the LV apex and base, the control group showed higher expression, although there was no statistical significance (Figure 6).

### Myocyte size

There were no significant differences in myocyte size among the three groups ([µm] CT: 11±2; UNI: 11±2; BIL: 12±1.5; *p*=0.9).

## DISCUSSION

Although there are numerous publications on the effects of sympathectomy in pathological scenarios, data on physiological models are rare (8,9). Therefore, we evaluated the physiological response of variables related to the autonomic nervous system, such as chronotropic properties at rest and exercise, cardiovascular autonomic modulation, myocardial and peripheral catecholamines, and beta-adrenergic myocardial receptors, following sympathectomy. The effects on ventricular function and myocyte size were also analyzed.

In the analysis of the QT interval, Yanowitz et al. (19) evaluated electrocardiographic changes in dogs following sympathectomy due to excision of the stellate ganglion and their stimulation by electrodes. QT prolongation was identified after right sympathectomy, and only a slight or no change after left sympathectomy. Following ganglion stimulation, the left side responded with an increase in QT interval, but no changes occurred on the right side. In the evaluation of QT interval in humans, Schwartz et al. (20) reported a decrease in QTc (corrected QT) 6 months after denervation of patients with long QT syndrome. Collura et al. (21) found no significant difference in the QT interval after left sympathectomy in patients with long QT syndrome and catecholaminergic polymorphic ventricular tachycardia. Our QT interval analysis showed no significant differences between groups. These findings, and also considering the nonuniformity of the literature, lead us to believe that sympathetic tone is not the main factor influencing this parameter.

In echocardiographic analysis of rats, Jiang et al. (9) showed an increase in systolic and diastolic volumes with a decrease in EF in the chemical sympathectomy group compared to the control group. Furthermore, Schlack et al. (22) showed that left sympathectomy acutely alters regional LV contractility, delaying the contraction and relaxation of the denervated area compared to that of the non-denervated area, leading to diastolic dysfunction. This same group repeated the study (23) in dogs with pacemaker-induced HF and showed an expressive worsening of diastolic and global LV function with a decrease in cardiac output. In humans, data from Lobato et al. (24) showed no significant differences in systolic and diastolic functions before and after sympathectomy by unilateral left or right ganglion block in patients without heart disease. Cruz et al. (25) reported a decrease in EF and diastolic change 6 months after sympathectomy for the treatment of hyperhidrosis. In our study, we found no significant difference in EF, probably due to the secondary hyperdynamic state and higher peripheral sympathetic activity, as corroborated by other findings (HR, catecholamine levels). Regarding left ventricular geometry, we found borderline differences (LVSD, *p*=0.06; LVDD, *p=*0.09), with larger diameters in the CT group compared to those in the UNI and BIL groups, which can also be inferred in the same way as EF (hyperdynamic state and higher peripheral catecholamine).

In this physiological setting, HR measurements at rest before the exercise were increased in the BIL group. Yoshimoto et al. (8) described a significant decrease in HR in relation to the sham group following bilateral sympathectomy. Our findings suggested that higher expression of β1 receptor in the BIL group (even with no statistical significance) and a higher level of peripheral catecholamine could be responsible for this increase. Regarding BP, Yoshimoto et al. (8) showed that there was no significant difference between groups, even after administration of atenolol. Moreover, Jiang et al. (9) reported significantly lower HR and MAP values following chemical sympathectomy with 6-hydroxydopamine compared to control rats. However, it should be noted that the chemical sympathectomy model with 6-hydroxydopamine leads to systemic denervation, including renal, by the peritoneal infusion of the drug. Perlini et al. (26) compared chemical sympathectomy with 6-hydroxydopamine against propranolol, doxazosin, and placebo in rats with LV hypertrophy by aortic banding. This study found no significant difference in HR between drugs or placebo. The banded animals had higher SBP and DBP compared to their controls in all three groups. In the chemical sympathectomy model with guanethidine, Julien et al. (27) found no significant changes in BP, only in BP variability, in which the maintenance of BP was attributed to the activation of the renin-angiotensin-aldosterone system. Our findings showed increased BP in the BIL group, which can also be inferred from an increased peripheral sympathetic tone found in the animals that underwent the procedure, as in the HR findings.

A study with dogs comparing right and left sympathectomy (26) demonstrated, at the peak physical exertion, that the left side had the highest HR, followed by the control, and then the right side. The right side had lower HR after the procedure at rest and at peak of exertion, in addition to a reduced ability to exercise. These findings are consistent with the expectations from a physiological point of view, considering that the right stellate ganglion predominantly innervates the atria and stimulates the sinus and atrioventricular node, whereas the left stellate ganglion predominantly innervates the ventricles by increasing the contraction force and consequently BP during stimulation. However, our study demonstrated an increase in the sympathetic activity in both sympathectomized groups, and the animals that were more tachycardic at rest demonstrated an increase in HR during exercise, again, most likely due to increased peripheral catecholamines.

In the evaluation of autonomic modulation, Jiang et al. (9) found lower HR variability in both the time domain (SDNN, SDANN, and RMSSD) and frequency (LF, HF, and LF/HF), suggesting that the rats in the chemical sympathectomy group had parasympathetic dominance. Our analysis demonstrated an increase in the parasympathetic activity suggested by the higher HF activity in the BIL and UNI groups. This effect in the UNI and BIL groups was attributed to a compensatory attempt of increased peripheral sympathetic tone.

Regarding the expression of beta receptors, in a publication that described the electrophysiological effects of bilateral sympathectomy in rats, Xie et al. (28) demonstrated a lower expression of β1 receptors by immunofluorescence in the sympathectomy group compared to the sham, inferring downregulation. In a canine model comparing control, chemical sympathectomy with 6-hydroxydopamine, and surgical sympathectomy, Valette et al. (29) demonstrated increased β-receptor expression in the two modes of sympathectomy evaluated using PET. It is expected that adrenoceptors would be upregulated by beta-blockers (except for those with intrinsic sympathomimetic activity that may lead to downregulation) related to the degree of selectivity (28). In cases of chronically sympathetic tonus increase, as in heart failure, the β-adrenoceptors would be expected to be downregulated. The literature is not uniform in the dosing methodology or sympathectomy procedure. Our findings suggest downregulation of β2, and even without statistical confirmation, upregulation of β1 in the BIL group, which may infer questions about the sensitivity of the receptors to different amounts of catecholamines. The BIL group had the highest expression of peripheral catecholamines, and responded with higher expression of β1 receptors, whereas the UNI group had a lower amount of peripheral catecholamines and expressed fewer β1 receptors; these findings imply a nonlinear relationship between catecholamine concentration and beta receptor expression.

Regarding catecholamines, Yoshimoto et al. (8) reported a significant decrease in the expression of myocardial catecholamines in the four cardiac chambers in bilateral sympathectomy compared to the sham group. However, the expression of peripheral catecholamines was not evaluated. Furthermore, Jiang et al. (9) reported a decrease in myocardial noradrenaline in the LV after chemical sympathectomy with 6-hydroxydopamine. An important finding in our study was the significant increase in peripheral catecholamines, corroborating the hypothesis of an extracardiac sympathetic compensatory pathway, and considering that myocardial catecholamines showed no significant difference between the groups. In a pathological scenario, as is the case for myocardial infarction, Zanoni et al. (30) found a protective effect of bilateral sympathectomy, possibly due to over stimulation of sympathetic tonus.

There are minimal data for tissue analysis after sympathectomy in nonpathological models. Jiang et al. (9) concluded that chemical sympathectomy induced degeneration of cardiomyocytes with necrosis and focal inflammatory infiltration, interstitial connective tissue hyperplasia, and collagen deposition. However, these findings may be related to the probable toxic effect of 6-hydroxydopamine on myocytes, as previously described (29). Our study exclusively analyzed myocyte size and found no significant difference.

## CONCLUSION

Our study showed that, despite unilateral or bilateral sympathectomy, the sympathetic tonus is evidenced by the increase of HR at rest and sustained in exercise, in addition to higher BP values at rest. This response is likely due to higher concentrations of peripheral catecholamines, suggesting possibility of an extracardiac sympathetic compensation pathway. We also observed a non-significant upregulation of β1 receptors in the BIL group, which may justify the increase in response to the higher circulating level of catecholamines, thus promoting higher HR and BP. Additional studies that overcome the limitations of the current proposal may help to confirm this hypothesis. It is advisable to await further clarification of the physiological impact of the sympathectomy before considering this procedure in clinical settings.

## AUTHOR CONTRIBUTIONS

Jordão MR contributed in conceptualization, data curation, investigation, methodology, writing-original draft, writing-review editing and formal analysis. Pessoa FG contributed in data curation, investigation, methodology, writing-original draft, writing-review editing and formal analysis. Fonseca KCB, Ribeiro ON and Irigoyen MC contributed in data curation, investigation and methodology. Zanoni F, Salemi VMC and Souza LE contributed in investigation, and methodology. Fernandes F contributed in conceptualization, investigation and project administration. Moreira LFP contributed in conceptualization, methodology and writing-original draft. Mady C contributed in conceptualization, writing-review editing and funding Acquisition. Felix José Alvarez Ramires FJA contributed in conceptualization, data curation, writing-original draft, writing-review editing, project administration, formal analysis and funding Acquisition.

## Figures and Tables

**Table 1:** Primers.

	Primer	Sequence	Reference
β1	Forward	5′CTGCTACAACGACCCCAAGTG3′	Sato et al. (16)
	Reverse	5′AACACCCGGAGGTACACGAA 3′	
β2	Forward	5′GAGCCACACGGGAATGACA 3’	Sato et al. (16)
	Reverse	5’CCAGGACGATAACCGACATGA3’	
GAPDH	Forward	5′ATGTATCCGTTGTGGATCTGAC3’	Bai and Meng (17)
	Reverse	5′CCTGCTTCACCACCTTCTTG3’	
β-actin	Forward	5’GTGCTATGTTGCCCTAGACTTCG3’	Yüzbasioglu et al. (18)
	Reverse	5’GATGCCACAGGATTCCATACCC3’	

**Table 2:** Echocardiogram.

	CT	UNI	BIL	*p*-value
LVSD (mm)	4.2±0.6	3.8±0.7	3.4±0.5	0.06
LVDD (mm)	7.6±0.7	7.1±0.7	7.1±0.7	0.09
LVEF (%)	81±6	80±10	87±4	0.18
LA (mm)	2.4±0.1	2.5±0.5	2.6±0.3	0.8
Sept (mm)	1.2±0.1	1.3±0.1	1.1±0.1	0.2
FW (mm)	1.1±0.1	1.2±0.2	1.2±0.2	0.85

CT: Control, UNI: Left unilateral sympathectomy, BIL: Bilateral sympathectomy, LVEF: Left ventricular ejection fraction, LVDD: Left ventricle diastolic diameter, LVSD: Left ventricular systolic diameter, LA: Left atria, Sept: Septum thickness, FW: Free wall thickness.

**Table 3:** Treadmill tests.

	CT	UNI	BIL	*p*-value
Treadmill test time (minutes)	9.8±2.7	10.0±2.0	8.1±1.8	0.26
Treadmill test maximal velocity (km/h)	1.2±0.3	1.2±0.2	1.0±0.2	0.32
Treadmill test distance (km)	0.13±0.06	0.13±0.04	0.09±0.03	0.35

BIL: Bilateral sympathectomy, CT: Control, UNI: Left unilateral sympathectomy.

**Table 4:** Blood pressure at rest.

	CT	UNI	BIL	*p*-value
Systolic blood pressure (mmHg)	130±9*	129±15**	144±8	* *p*=0.02 ** *p*=0.04
Diastolic blood pressure (mmHg)	98±10	98±15	110±7	0.11
Mean arterial pressure (mmHg)	109±9	108±14	121±7	0.07

BIL: Bilateral sympathectomy, CT: Control, UNI: Left unilateral sympathectomy.

* *p*= 0.02 BIL *vs* CT

** *p*=0.04 BIL *vs* UNI.

**Table 5:** Blood pressure immediately after exercise.

	CT	UNI	BIL	*p*-value
Systolic blood pressure (mmHg)	125±14	132±11	143±10*	<0.05
Diastolic blood pressure (mmHg)	94±14	98±11	110±7*	0.04
Mean arterial pressure (mmHg)	104±13	109±10	121±8*	0.03

BIL: Bilateral sympathectomy, CT: Control, UNI: Left unilateral sympathectomy.

* BIL *vs* CT.

**Table 6:** Autonomic modulation results.

	CT	UNI	BIL	*p*-value
PI SD	10±5	11±5	10±5	0.93
PI VAR	127±111	143±91	118±126	0.93
SBP VAR	44±31	95±113	54±30	0.80
LF	4.49±3.08	7.24±1.81	9.92±8.87	0.34
LF α index	1.59±1.42	1.03±0.55	1.04±0.51	0.51

BIL: Bilateral sympathectomy, CT: Control, LF: Low frequency, LF α index: Low frequency a α index, PI SD: Pulse interval standard deviation, PI VAR: Pulse interval variance, SBP VAR: Systolic blood pressure variance, UNI: Left unilateral sympathectomy.
